# New Pneumococcal Carriage Acquired in Association with Acute Respiratory Infection Is Prone to Cause Otitis Media

**DOI:** 10.1371/journal.pone.0156343

**Published:** 2016-06-03

**Authors:** Kari Auranen, Ritva Syrjänen, Tuija Leino, Terhi Kilpi

**Affiliations:** 1 Department of Health Protection, National Institute for Health and Welfare, Mannerheimintie 166, 00300 Helsinki, Finland; 2 Department of Mathematics and Statistics, 20014 University of Turku, Turku, Finland; 3 Department of Health Protection, National Institute for Health and Welfare, Finn-Medi I, Biokatu 6, 33520 Tampere, Finland; Instituto Butantan, BRAZIL

## Abstract

For considering vaccine-prevention of pneumococcal acute otitis media (PncAOM), relationships between pneumococcal carriage, respiratory infection and PncAOM need to be understood. We analyzed nasopharyngeal samples collected from 329 unvaccinated Finnish children aged 2–24 months at scheduled visits and at visits during respiratory infection in 1994–97. We assessed temporal associations of respiratory infection with pneumococcal acquisition and whether PncAOM hazard depends on the relative timing of acquisition and the infection onset. The data comprised 607 person-years of risk-time for acquisition, 245 person-months of concurrent respiratory infection and carriage, and 119 episodes of PncAOM. The acquisition hazard was 3-fold in the month preceding respiratory sickness (hazard ratio, HR 3.5, 90% credible interval CI 2.9, 4.1) as compared to acquisition in healthy children. Moreover, the PncAOM hazard was markedly higher (HR 3.7, 90% CI 2.4, 5.3) during the first month of carriage acquired around the acute phase of respiratory infection (between 1 month before and 1 week after the sickness onset), as compared to carriage acquired later during sickness. The high proportion (76%) of PncAOM events occurring within 1 month of acquisition was due to frequent acquisition being associated with respiratory infection as well as the susceptibility of such acquisition to cause otitis media.

## Introduction

*Streptococcus pneumoniae* belongs to the normal bacterial flora in the human upper respiratory tract. The usually asymptomatic carrier state only occasionally progresses to illness. The most common manifestation of pneumococcal illness in developed countries is acute otitis media (AOM). Both pneumococcal carriage and illness are associated with concurrent or preceding respiratory infection [1–7].

We have previously reported a gross difference in the frequency of pneumococcal carriage between age-based samples and samples collected during symptoms of respiratory infection in the same cohort of children [8]. As pneumococcal carriage does not cause symptoms, such association between carriage and illness could result from simultaneous exposure to viral and bacterial pathogens. Viral respiratory infection may also directly enhance pneumococcal carriage acquisition due to facilitated bacterial adherence in the nasopharynx through upregulated expression of adhesion molecules [9].

Nasopharyngeal carriage of pneumococci and concurrent viral respiratory infection are important factors in development of pneumococcal AOM (PncAOM). Homologous pneumococci are invariably found in the middle ear and the nasopharynx during PncAOM [10], indicating necessity of a pneumococcal reservoir in the nasopharynx underlying PncAOM. In addition, molecular techniques have shown that almost every child with AOM also has viral infection [11–13].

Gray et al. [14] argued most PncAOM cases occur within 1 month after pneumococcal acquisition. Sleeman et al. [15] showed an association between acquisition and general practitioner consultations due to potentially pneumococcal infection. The proportion of PncAOM following a recently acquired episode of pneumococcal carriage depends on how strongly pneumococcal acquisition is associated with respiratory infection and how susceptible these acquisitions are to proceed to cause PncAOM. Disentangling such effects requires longitudinal data and availability of specific AOM etiology.

Based on the FinOM Cohort Study, we have previously reported a clear association of the PncAOM risk and recent carriage acquisition [16]. However, the rate of PncAOM was not quantified per at-risk time and the relative timing of respiratory infection, pneumococcal acquisition and PncAOM was not assessed. We now revisit the FinOM Cohort data by analysing pneumococcal acquisition and emergence of PncAOM as processes in continuous time. We address two specific questions: How does pneumococcal acquisition increase from its baseline level during respiratory infection? During concurrent pneumococcal carriage and respiratory infection, how does the PncAOM hazard depend on the timing of carriage acquisition with respect to the infection onset? We found that a high proportion of PncAOM events occurs within one month of pneumococcal acquisition. Moreover, this follows from the relatively high proportion of pneumococcal acquisition being associated with the acute phase of respiratory infection and the susceptibility of these acquisitions to lead to otitis media.

## Materials and Methods

### Data and Definitions

#### Age-based and sick visits

The FinOM Cohort data have been described in detail elsewhere [8, 17]. Briefly, 329 children not vaccinated against pneumococci were followed from 2 until 24 months of age in 1994–1997. Serotype/group-specific pneumococcal carriage was recorded using nasopharyngeal swab samples at 10 age-based visits at 2, 3, 4, 5, 6, 9, 12, 15, 18 and 24 months of age. In addition, parents were asked to bring their child to a study clinic whenever the child needed medical care for acute respiratory infection, especially under AOM suspicion. At these sick visits, pneumococcal carriage was recorded by nasopharyngeal aspirate (NPA) samples and AOM was diagnosed by pneumatic otoscopy and clinical signs of acute infection [8]. Whenever AOM was diagnosed, its etiology was investigated by collecting a middle ear fluid (MEF) sample for bacterial culture. Pneumococcal presence in the MEF from one or both ears is called PncAOM.

Samples were cultured, pneumococci identified and serotypes determined using standard techniques [8]. Because the sensitivity of sampling and culture is not 100% and carriage is considered a necessary prerequisite for bacterial presence in the middle ear [10, 18], a negative or missing NPA, or NPA with a different serotype from that in a concurrent MEF sample was imputed with the MEF serotype. In addition, any non-carriage sample was interpreted as false negative and imputed with positive carriage if the child was on antibiotic medication and both the previous and next samples of the same serotype were <62 days from the initially negative sample. To identify pneumococci, 4 colonies were tested, a method now known to be insensitive in identifying multiple carriage [19]. If more than 1 serotype was isolated in 1 sample, the concurrent MEF serotype was retained. Otherwise the newer serotype was retained, or if more than 1 new serotype was found concurrently, a random one.

#### Episodes of carriage and non-carriage

Episodes of carriage/non-carriage were defined as series of observations of the same serotype/non-carriage at consecutive visits. For each episode, the onset time was defined as the midpoint between the first observation of the serotype/non-carriage during the episode and the previous visit. Likewise, each episode ended midway between the last observation during the episode and the following visit. If the episode ended at the child’s last observation, the statistical model treated this information by ‘censoring’ the episode (in the standard statistical sense), i.e. used the information that the episode lasted at least up to the time of censoring. The same applied if there were ≥62 days since the child’s previous sample. The next episode’s onset was then considered unknown. The child was assumed to be a non-carrier at birth. Episodes of carriage and non-carriage were used in exploratory analyses and in defining risk episodes for PncAOM (see below). In the episode-based analyses, pneumococcal acquisition was identified as any carriage episode with known onset time.

#### Sick episodes

**Fig 1:**
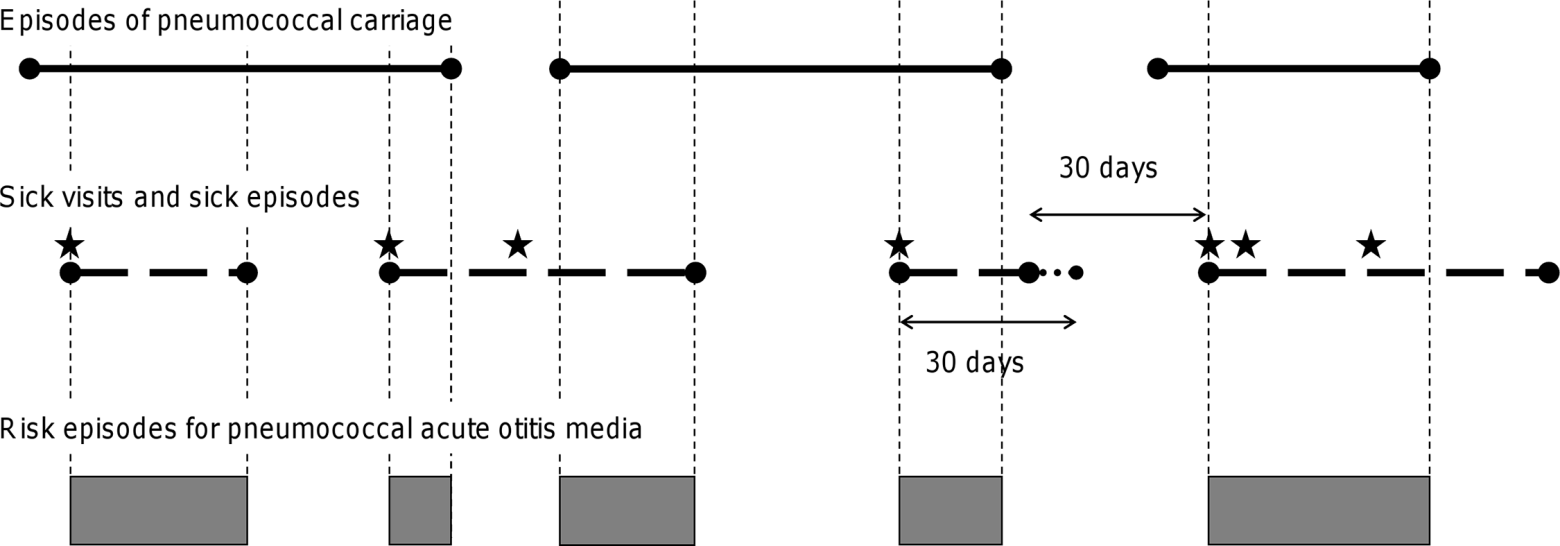
Risk episodes for pneumococcal acute otitis media (PncAOM), FinOM Cohort Study, Finland, 1994–97. Solid lines: Episodes of pneumococcal carriage defined using nasopharyngeal samples obtained <62 days apart. Dotted lines: Sick episodes defined to start if the child visited the study clinic because need of medical care for respiratory infection (stars), and if >30 days had elapsed since previous such sick visit, and to last for 30 days after the last sick visit during the sick episode. Bars: Risk episodes for PncAOM, defined as overlapping periods of carriage and sick episodes.

A new sick episode was taken to start at a sick visit if >30 days had occurred since the child’s previous sick visit, and end 30 days after the sick episode’s last sick visit or 30 days before the next sick episode onset, whichever occurred earlier (Fig 1). With modern methods for detection of a wide panel of viruses, virtually all young children with symptoms of respiratory infection can be shown to harbour viruses in their nasopharynges [13]. The sick episodes are thus interpreted as fixed episodes of viral respiratory infection.

#### Risk episodes

Risk episodes for PncAOM were defined as overlapping periods of carriage episodes and sick episodes (Fig 1). For each risk episode, it was required that the MEF sample at the first potential PncAOM be available and the carriage onset be known, except when the carriage onset was >30 days before the risk episode onset and the presence of any previous sickness during the carriage episode could be inferred from the data.

### Statistical analysis

#### Pneumococcal acquisition

As an exploratory analysis, a non-parametric (Nelson-Aalen) estimate of the cumulative hazard of pneumococcal acquisition in children with at least 1 sick episode was determined. For each sick episode, the at-risk time for acquisition started at the end of the previous sick episode or, if there was no such episode, at the onset of follow-up, and lasted until the sick episode’s end.

Based on the episodes of carriage and non-carriage, crude estimates of pneumococcal acquisition hazards were calculated. To adjust for the effect of concurrent carriage [20], acquisition was considered separately for episodes of non-carriage (acquisition in non-carrying children) and carriage (acquisition of new serotypes in carrying children). Based on the non-parametric exploratory analysis, the time at-risk for acquisition was stratified into 3 categories according to proximity to sick episode onsets.

The hazards of pneumococcal acquisition and clearance from all age-based and sick visit samples were estimated using a Markov transition model (S1 Appendix). For any child, the 31 possible states included `non-carrier' or `carrier' of any of the 30 serotypes in the data. Based on a change-point in the age-specific carriage prevalence [8], the hazards were stratified into 2 age groups (<12 and ≥12 months). The same hazards of acquisition and clearance were assumed for each serotype. A carrying child was assumed to experience 50% reduction in the acquisition hazard as compared to a non-carrying child of the same age [21]. The acquisition hazards were stratified according to the 3 categories. The Markov model used all data, and acquisition events were implicitly defined through the model.

The model fit was assessed by comparing model-based predictions of the carriage prevalence at age-based and sick visits to the actual observations. In addition, the predicted prevalence in children without any sick episodes was compared with the observed prevalence at age-based visits made more than 30 days before the first sick visit of the child.

#### Emergence of PncAOM

For each risk episode, the first PncAOM was considered. The child entered risk for PncAOM at the risk episode onset and exited at the first PncAOM or termination of episode whichever occurred first (Fig 1). In the exploratory analysis, Nelson-Aalen estimates of the cumulative PncAOM hazard were determined. Because of the *a priori* hypothesis that current duration of carriage affects PncAOM hazard, time since the onset of the underlying carriage episode was chosen as the principal time scale. The exploratory analysis was used to classify risk episodes into 3 strata by the timing of carriage acquisition with respect to the sick episode onset, so that the PncAOM hazard was almost constant within each stratum.

PncAOM hazards were estimated in the 3 strata of risk episodes using Poisson regression, with adjustment for age (<6, 6–11, 12–17, ≥18 months at the risk episode onset), previous homologous carriage (yes/no), and previous homologous PncAOM (yes/no). To remove the potential impact of repeated sick episodes during the same carriage episode a separate regression analysis was conducted involving only the first sick episodes during carriage. All parameter estimates are presented as Bayesian posterior means and 90% probability (credible) intervals, CI [22].

### Ethical Review

Written informed consent was obtained from the parents or guardians. The study protocol was approved by the Ethics Committees of the National Institute for Health and Welfare, Department of Social and Health Care of Tampere City, and Tampere University Hospital.

## Results

### Carriage at Age-based and Sick Visits

**Fig 2:**
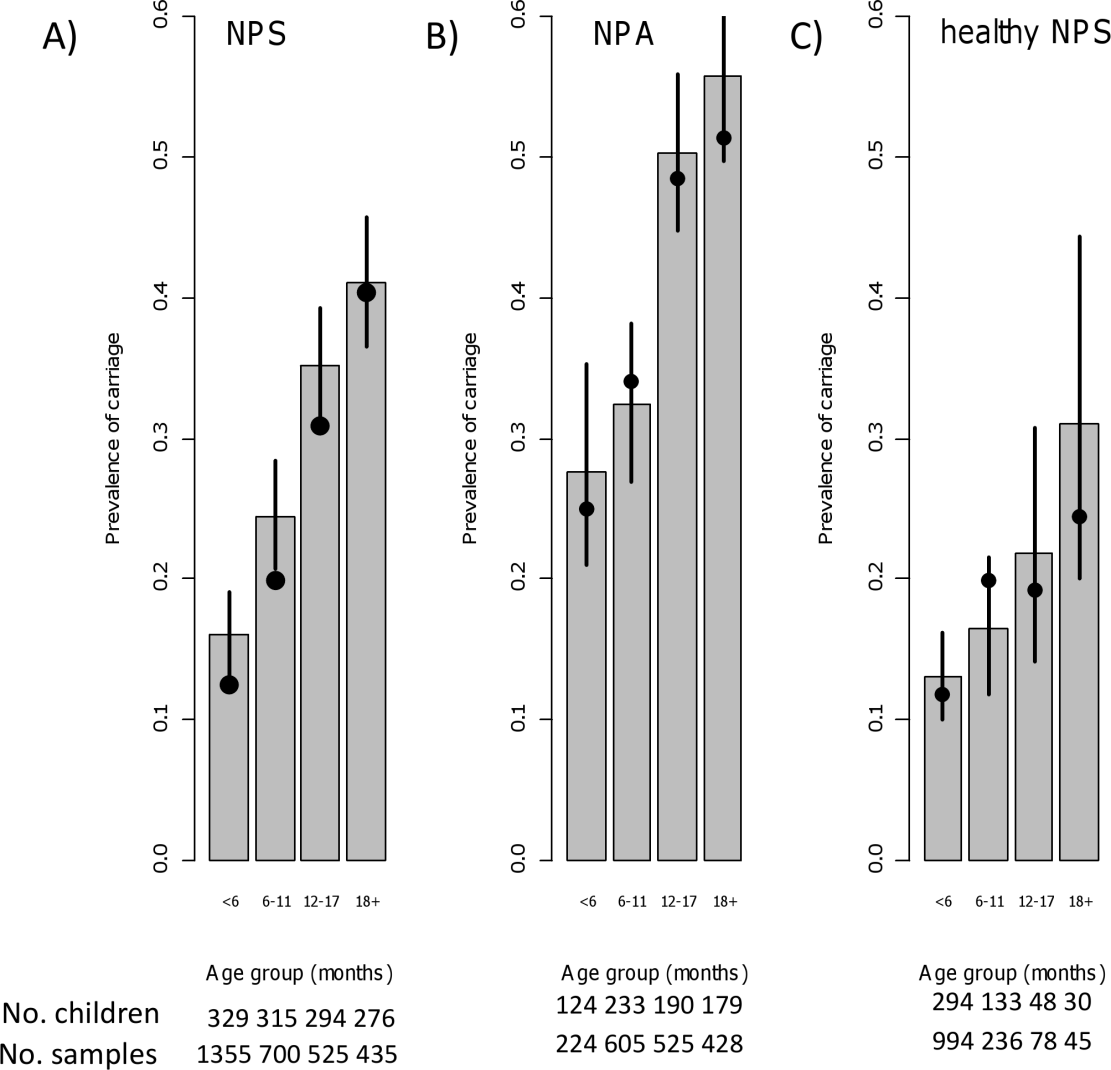
The observed and predicted prevalence of pneumococcal carriage during health and respiratory infection, FinOM Cohort Study, Finland, 1994–1997. Panel (A): Age-based samples (nasopharyngeal swab samples, NPS); Panel (B): Sick visit samples (nasopharyngeal aspirates, NPA); Panel (C): Age-based samples (NPS) collected more than 30 days prior to any of the sick episodes in the child. In each panel, the prevalence is shown for 4 age groups. The observed values are shown marked with black dots. The bar heights indicate the predicted prevalence values, as based on the estimated model parameters. The vertical lines show the 90% predictive intervals. The numbers of children and the numbers of samples in the four age groups by visit sample type (NPS, NPA, healthy NPS) are indicated at the bottom of the figure.

There were 3026 age-based and 1897 sick visits, resulting in 3015 age-based and 1782 sick visits with serotype/group-specific information (S1 Fig). The carriage prevalence increased with age in both visit types (Fig 2, panels A and B, dots). In children aged <6 months, the carriage frequency was double when comparing the sick and age-based visits (25% vs. 12%). The difference between the 2 visit types decreased with age, being only moderate in children aged ≥18 months (51% vs. 40%).

After excluding 731 samples collected ≥62 days from any other sample, 2330 age-based and 1736 sick visit samples were available to define serotype-specific episodes of carriage (N = 585) (S1 Fig) and non-carriage (N = 828). The age-specific carriage prevalence was similar in the excluded samples and those used to define the episodes. Altogether 228 children had at least 1 carriage episode (median 2; range 1–9).

### Sick Episodes

Of all 1897 sick visits, 1146 started a sick episode. The median number of sick episodes was 4 (range 1–10). Of the sick episodes, 346 (30%) ended at exactly 30 days before the next sick episode’s onset. The sick episodes constituted 17% of the total follow-up time of 607 person-years in the 329 children.

### Pneumococcal acquisition in relation to respiratory infection

**Fig 3:**
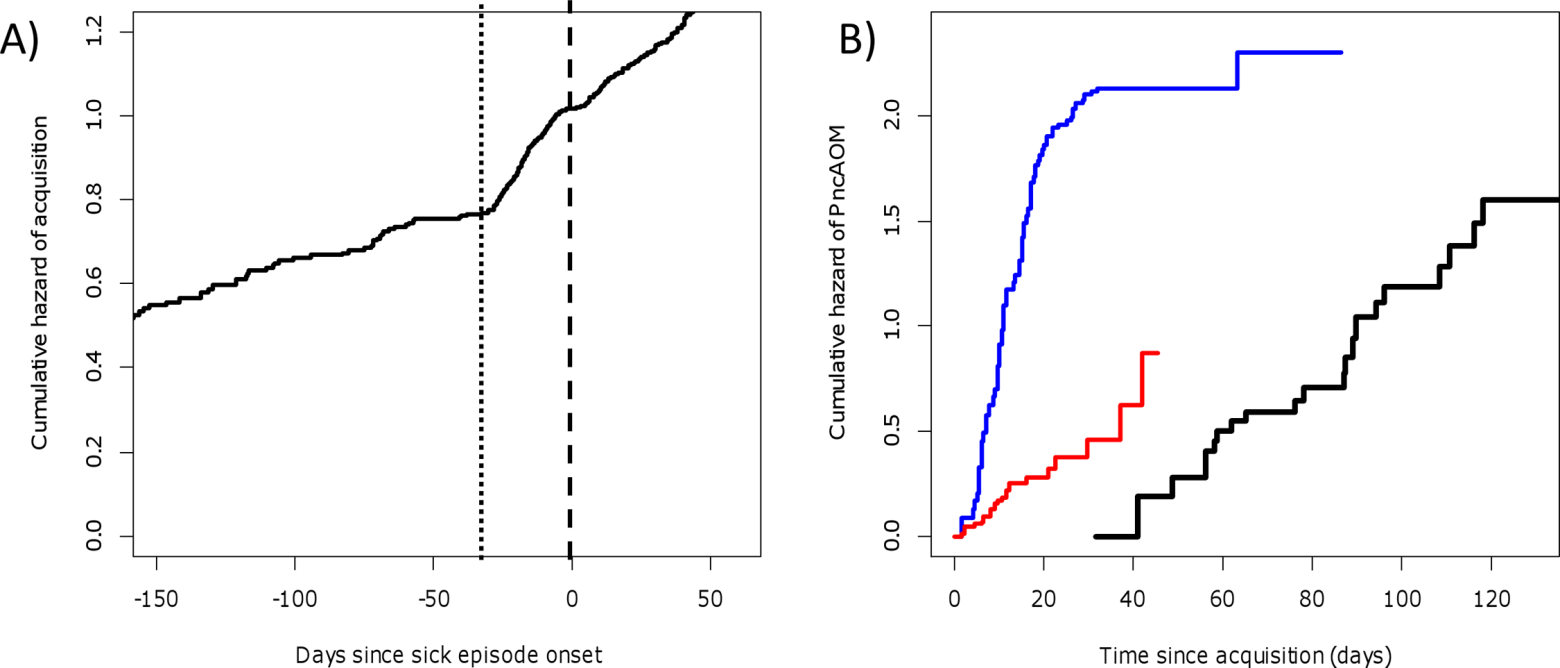
Panel (A): Acquisition of pneumococcal carriage and respiratory infection. The figure presents the Nelson-Aalen estimate of the cumulative rate of pneumococcal acquisition in the 286 children who had least 1 sick episode (altogether 1146 sick episodes). For each sick episode, the at-risk time for carriage acquisition started at earliest at the end of the preceding sick episode and lasted until the end of the sick episode, with the total at-risk time of 247 person-years. The time origin is at the sick episode onset. Times of acquisition events (N = 394) were identified by the time of carriage episode onset if known by our carriage episode definition. Within the time span shown in the Figure, 362 acquisition events occurred. **Panel (B):** Hazard of pneumococcal otitis media. The cumulative hazard of pneumococcal otitis media (PncAOM) during the risk episodes (N = 439) is presented in 3 strata, defined by the timing of the underlying carriage acquisition in relation to the sick episode onset: 1 month before = black line (124 risk episodes); within 1 month before or 1 week after = blue line (204 risk episodes); more than 1 week after = red line (111 risk episodes). S1 Fig shows how the risk episodes were defined based on the sick episodes and episodes of pneumococcal carriage. The numbers of PncAOM events and person-times in the three strata are given in Table 1.

Based on data from all 286 children with at least 1 sick episode, the non-parametric estimate of the pneumococcal acquisition hazard was clearly higher during 1 month preceding the sick episode onset and remained elevated throughout the sick episode, when compared to the acquisition hazard more than 1 month before the sick episode onset (Fig 3A). According to this exploratory analysis, the at-risk time for acquisition was stratified into 3 categories according to time spent during pre-sickness (during 1 month preceding any sick episode onset), during sickness (the sick episode), and during health (all other times) in all further analyses.

**Table 1:** Hazard of Pneumococcal Otitis Media by Age Group and Timing of Carriage Acquisition With Respect to Sick Episode Onset, FinOM Cohort Study, Finland, 1994–1997.

	Time of carriage acquisition in relation to the sick episode onset												$\tilde{\lambda }$^f^
Age group^a^	>1 month before^b^				≤1 month before or ≤1 week after^c^				>1 week after^d^				
	*N*	*Y*	$\hat{\lambda }$^e^	90%CI	*Y*	*N*	$\hat{\lambda }$^e^	90%CI	*N*	*Y*	$\hat{\lambda }$^e^	90%CI	
**<**6	4	5.5	0.73	0.32,1.67	7	6.3	1.11	0.60,2.07	1	3.2	0.31	0.06,1.63	0.80
6–11	13	23.1	0.56	0.36,0.89	19	10.6	1.80	1.23,2.62	6	13.3	0.45	0.23,0.88	0.81
12–17	3	18.5	0.16	0.06,0.42	28	13.0	2.15	1.57,2.93	13	22.9	0.57	0.36,0.90	0.81
≥18	3	4.1	0.74	0.28,1.90	13	9.2	1.41	0.89,2.22	6	13.1	0.46	0.23,0.90	0.83
Total	23	51.1	0.45	0.32,0.63	67	39.1	1.71	1.40,2.09	26	52.5	0.50	0.36,0.68	0.81

Abbreviations: CI, Bayesian posterior probability (credible) interval; mo, months; *N*, number of PncAOM events; PncAOM, pneumococcal acute otitis media; *Y*, person-time (months) at risk during the risk episodes (overlapping periods of carriage and sickness).

^**a**^ Age group at the onset of the risk episode (months).

^**b,c,d**^ The at-risk time was considered in 3 strata (see text). These correspond to the 3 slopes in Fig 3B: ^a^ black curve; ^b^ blue curve (PncAOM hazard for the first 30 days of carriage, during which 67 of 70 PncAOMs occurred. Thereafter, there were only 3 PncAOM events from a person-time of 34.7 months, corresponding to PncAOM hazard of 0.09 per month); ^c^ red curve.

^**e**^ Crude estimates of the hazard of PncAOM, *λ*(per month), were calculated as the number of PncAOM events (*N*) divided by the corresponding person-time (*Y*).

^**f**^ Crude overall estimates of PncAOM hazard

Table 2 stratifies the observed acquisition events and person-times by age, current carriage (non-carrier/carrier), and proximity to sick episode onset (during health/pre-sickness/sickness). The unadjusted overall acquisition hazard in a non-carrying child was 5.4 (= 140/509/(67/1308)) times higher during pre-sickness than during health. During sickness, the acquisition hazard continued to be elevated when comparing to health in non-carrying children (HR 3.1). These exploratory comparisons of acquisition hazards during sickness and health are confounded because of the low hazard in children <6 months old who nevertheless provided most directly observed acquisition events during health.

**Table 2:** Observed Numbers of Acquisition Events and Person-Time, Stratified by Age Group, Current Status of Carriage, and Proximity to Sick Episode Onset, FinOM Cohort Study, Finland, 1994–1997.

Age^a^	Non-carrier^b^						Carrier^b^					
	Health^c^		Pre-sickness^c^		Sick episode^c^		Health^c^		Pre-sickness^c^		Sick episode^c^	
	*N/Y*	$\hat{\lambda }$	*N/Y*	$\hat{\lambda }$	*N/Y*	$\hat{\lambda }$	*N/Y*	$\hat{\lambda }$	*N/Y*	$\hat{\lambda }$	*N/Y*	$\hat{\lambda }$
<6	65/1206.0	0.05	47/248.5	0.19	18/218.3	0.08	4/145.1	0.03	9/69.3	0.13	9/52.7	0.17
6–11	1/52.2	0.02	39/130.0	0.30	25/180.1	0.14	0/20.5	0.00	14/71.6	0.19	13/83.3	0.16
12–17	1/39.3	0.03	43/94.6	0.45	33/116.8	0.28	1/25.3	0.04	14/87.8	0.16	18/106.9	0.17
≥18	0/10.2	0.00	11/35.5	0.31	14/58.6	0.24	0/8.4	0.00	12/45.9	0.27	13/79.0	0.16
Total	67/1307.5	0.05	140/508.5	0.28	90/573.8	0.16	5/199.3	0.03	49/274.6	0.18	53/321.8	0.16

Abbreviations: *N*, the observed number of pneumococcal acquisition events, based on the episodes of carriage and non-carriage with known onset times (for definition, see text); *Y* = person-time (months), based on all episodes of non-carriage (non-carriers) or carriage (carriers); $\hat{\lambda }$, a crude estimate of hazard of pneumococcal acquisition, $\hat{\lambda }$ = *N/Y* (per month), was calculated as the number of acquisition events (*N*) divided by the corresponding person-time *Y* (months).

^**a**^ Age group (in months) at the beginning of the episode (of non-carriage or carriage).

^**b**^ Non-carrier refers to episodes of non-carriage; an acquisition event in a non-carrier refers to the onset of a carriage episode; carrier refers to episodes of carriage, an acquisition event in a carrier refers to the onset of a carriage episode of another serotype.

^**c**^ Episodes of non-carriage and carriage are divided into 3 categories: health/pre-sickness/sick episode (for details, see text).

Based on the Markov transition model and all data from the 329 children, Table 3 presents the estimated hazards of pneumococcal acquisition and clearance. The acquisition hazards were 0.08 (90% CI 0.07, 0.09) and 0.18 (90% CI 0.16, 0.21) per month in healthy non-carrying children aged <12 and ≥12 months, respectively. The hazards of clearing carriage were similar in both age groups (0.37 per month 90% CI 0.32, 0.42, and 0.38, 90% CI 0.34, 0.42). These correspond to median carriage durations of 1.9 months with overlapping 90% CIs (1.7, 2.2 and 1.7, 2.1). The acquisition hazard was 3.5 times higher during pre-sickness as compared to health (HR 90% CI 2.9, 4.1). The hazard continued to be elevated during sickness (HR 2.2, 90% CI 1.9, 2.7).

**Table 3:** Hazards of Pneumococcal Acquisition and Clearance, FinOM Cohort Study, Finland, 1994–1997.

Parameter^a^	Estimate^b^	90% CI
Hazard of acquisition in a healthy non carrying child (per month)		
age <12 months	0.077	0.067,0.088
age ≥12 months	0.183	0.159,0.207
Relative hazard of acquisition		
presickness vs. health	3.5	2.9,4.1
sickness vs. health	2.2	1.9,2.7
Hazard of clearance (per month)		
age <12 months	0.37	0.32,0.42
age ≥ 12 months	0.38	0.34,0.42

Abbreviation: CI, Bayesian posterior probability (credible) interval

^**a**^ The hazards of acquiring and clearing pneumococcal carriage were estimated from all age-based (N = 3015) and sick visit samples (N = 1782) using a Markov transition model (Web Appendix). The hazard of acquisition refers to the overall pneumococcal acquisition for all 30 serotypes/groups in the data.

^**b**^ The Bayesian posterior mean estimate.

Fig 2 presents model predictions of pneumococcal carriage prevalence at the age-based (panel A, bars) and sick visits (panel B, bars). The agreement with the observations is good, despite a slight tendency to overestimation at age-based sampling. The predicted prevalence ratio comparing sick visit and age-based samples decreased from 1.7 in the youngest to 1.4 in children ≥12 months old. Fig 2 (panel C, bars) shows the model-based prediction of the age-specific prevalence of carriage assuming absence of any respiratory infections (i.e. sick episodes). For comparison, the prevalence in a subset of the data is shown, based on all age-based samples before which the child had had any sick episodes (panel C, dots).

### PncAOM in relation to pneumococcal acquisition and respiratory infection

There were 585 carriage episodes, producing 603 overlapping periods of carriage and sick episodes with 166 PncAOM events (S1 Fig). Eventually, 439 risk episodes (total duration 245 months) and 119 PncAOM events remained in the analysis. Altogether 171 children (52%) had at least 1 risk episode (median 2, range 1–9). These children were more often boys (61% vs. 44%) and had more often older siblings (64% vs. 40%).

Of all risk episodes, 27% (119/439) led to PncAOM in altogether 82 children (26%, 28%, 29%, and 23% in children aged <6, 6–11, 12–17, and ≥18 months, respectively). Of the 119 PncAOM events, 76% (91/119) occurred within 1 month after carriage acquisition.

Fig 3B shows the cumulative PncAOM hazard in 3 strata defined by the timing of carriage acquisition with respect to the sick episode onset (black/blue/red lines corresponding to acquisition >1 month before/within 1 month before or within 1 week after/>1 week after). The break points were chosen so that the hazard was relatively constant within each stratum. Of the 119 PncAOM events, 70 were due to carriage acquired around the sick episode onset. In this stratum (blue), the PncAOM hazard during the first month of carriage was notably higher than the hazards during risk episodes for which carriage had started earlier (black) or only later during sickness (red). Only 3 of the 70 PncAOM events from risk episodes with carriage acquisition around the sick episode onset occurred more than 1 month after acquisition, resulting in a very low PncAOM hazard in this period. Consequently, for risk episodes with carriage acquisition around the sickness onset, the Pnc AOM hazard was estimated only for the first month since acquisition, leaving 116 (= 67+49) PncAOM events for the Poisson regression model.

Table 1 stratifies the person-time and Pnc AOM events during the 439 risk episodes by age group and the 3 risk episode strata. Based on the Poisson model, the hazard was markedly higher during the first month of carriage if acquired around (i.e., within 1 month before or 1 week after) the sickness onset, as compared to risk episodes for which carriage was acquired either later during sickness (HR 3.7, 90% CI 2.4, 5.3) or more than 1 month before sickness (HR 4.9, 90% CI 2.9, 7.7). Accordingly, of the 116 PncAOM events, 67 (58%) were due to carriage episodes acquired around the sickness onset although this time window constituted only 23% (57/245) of the total follow-up during the risk episodes. Previous homologous carriage protected against PncAOM (HR 0.59, 90% CI 0.36, 0.90). Previous homologous AOM was not significantly associated with PncAOM hazard.

For a high proportion (91/124) of risk episodes for which carriage had started more than 1 month before the sickness onset, the child had had at least 1 earlier sick episode during the same carriage episode. The PncAOM hazard during the 91 repeated, i.e. second or later sick episodes during the same carriage episode was significantly smaller than the hazard during the 33 first sick episodes during carriage (0.34 vs. 0.78 per month, HR 0.49, 90% CI 0.22, 0.91). Of the 33 first risk episodes, 26 occurred in young children aged <12 months. By contrast, of the 91 repeated sick episodes, 40 were from children <12 months. The majority of the repeated sick episodes (66/91) occurred in children age at the age most susceptible to infections, i.e. 6–17 months.

To remove the effect of repeated sickness during carriage, we next compared PncAOM hazards between the 3 strata based solely on the first sick episodes of carriage (N = 348) (S1 Table). In this analysis, the ratio of PncAOM hazards, comparing risk episodes for which carriage was acquired around the sickness onset with those for which carriage was acquired later during sickness remained high (HR 3.7, 90% CI 2.5, 5.4). However, the ratio was smaller when comparing risk episodes for which carriage had been more than 1 month before (HR 2.3, 90% CI 1.2, 4.2). Previous homologous carriage decreased the PncAOM hazard (HR 0.49, 90% CI 0.25, 0.81). Previous homologous PncAOM was not associated with PncAOM hazard.

### Sensitivity analysis

We re-analyzed the data by censoring episodes of carriage/non-carriage if there was ≥45 days since the child’s previous sample. The acquisition hazard presented a sharper increase of shorter duration preceding the sickness onset (S2 Fig). Based on 328 risk episodes, differences in the PncAOM hazard between the 3 strata were even sharper than in the base-case analysis.

## Discussion

This study corroborates the earlier assertion that recently acquired pneumococcal colonization is prone to cause otitis media [14, 16]. Of the first PncAOM events during new episodes of respiratory infection, 76% (91/119) occurred within 1 month after pneumococcal acquisition. In addition, we asked whether this finding is rather explained by pneumococcal acquisition being in temporal association with respiratory infection or whether recent acquisitions *per se* poses an independent risk. We found that the PncAOM hazard does not depend on recent acquisition as much as on the association of acquisition with concurrent respiratory infection. In particular, 74% (67/91) of PncAOM events occurring within 1 month after acquisition were due to acquisition in temporal vicinity with the acute phase of respiratory infection. This phenomenon in turn was explained by the elevated rate of pneumococcal acquisition associated with the acute phase and the susceptibility of these acquisitions to lead to PncAOM. Importantly, even if new acquisition were relatively common after the acute phase of respiratory infection, the PncAOM hazard was significantly smaller than during the acute phase.

The pneumococcal acquisition hazard was 3-fold in the month preceding a new episode of respiratory infection as compared to health. A model assuming the same relative increase in acquisition across all age groups could explain the decreasing excess risk of pneumococcal carriage with age (Fig 2A and 2B). The prevalence ratio (sickness vs. health) was largest in the youngest because of the lower baseline prevalence during health. In absence of respiratory infection, carriage prevalence would increase only moderately with age (Fig 2C).

The estimated hazard of clearing pneumococcal carriage implied a median duration of approximately 2 months, in agreement with earlier estimates of the average duration carriage in young children [23,24]. This median duration means that long-lasting carriage episodes are not rare.The high proportion of newly acquired carriage underlying PncAOM is therefore not obvious.

The PncAOM risk in the FinOM Cohort children peaked at 1 year of age (17). Of the 119 first PncAOM events during the risk episodes 71% occurred between 6 and 18 months. However, when considered by risk episode or person-time at risk (Table 1, last column), the PncAOM risk did not vary with age, implying that the age-specific PncAOM incidence is largely modulated by the incidence of respiratory infection.

During concurrent carriage and respiratory sickness, the PncAOM hazard was highest during the first month of carriage if acquired in temporal association with respiratory infection. Comparing to carriage acquired after the acute phase of infection, the hazard ratio was large (HR 3.7, 90% CI 2.4, 5.3), and even larger when comparing to carriage acquired more than 1 month before sickness (HR 4.9, 90% CI 2.9, 7.7). In the latter comparison, however, most at-risk time in the group who had acquired carriage more than 1 month before sickness accrued from children who had been previously sick during the same long carriage episode. When the analysis was restricted to the first sickness during carriage, there was a smaller albeit significant difference in the PncAOM hazards between carriage acquired around the sickness onset and carriage acquired more than 1 month before sickness (HR 2.3, 90% CI 1.2, 4.2). There remains a potential source of bias due to more readily detecting long symptomless carriage episodes in the youngest children with the most frequent sampling.

Uneven sampling posed a potential caveat for the episode-based analysis of pneumococcal acquisition. Episodes of carriage were more readily identified in young children with more frequent schedule of age-based visits, or in children with many sick visits. In the episode-based analysis, the acquisition hazard in healthy children derived mainly from the very young whereas in the older children acquisition was mostly observed in association with sickness. Nevertheless, the Markov transition model used all available carriage data to estimate the hazards of acquisition and their dependence on sickness.

Although the youngest children were overrepresented among the age-based samples, most person-time at risk for PncAOM accumulated in children at the infection-prone age of 6–18 months. Therefore, it is likely that most potential risk episodes were identified due to the active follow-up, in which 81% of all sickness-related visits took place in the study clinic [8]. Moreover, since respiratory infection is necessary for the development of otitis, our analysis of PncAOM hazards should not suffer from selection towards sick children or periods of sickness.

Our interpretation of the data is that viral infection caused the symptoms that led to the sick visit and at the same time made the child more susceptible to pneumococcal acquisition. We cannot definitely rule out the possibility that pneumococcal acquisition caused the symptoms leading to the sick visit. However, because it takes at least some days from infection to symptoms and symptoms usually started several days before the sick visit, viral acquisition had probably occurred at least 1 week before the sick episode onset. Although the time of increased acquisition was initially found to start 1 month earlier, the sensitivity analysis showed this time window depends on the definition of carriage episodes and could be shorter (S2 Fig). There is ample evidence from both in vitro and experimental studies that viral infection enhances pneumococcal binding to respiratory cells, predisposing to pneumococcal acquisition [9, 25–27]. We consider it plausible that mucosal changes caused by viral infection predisposed the child for pneumococcal adhesion.

Respiratory infection contributes to development of otitis media. Inflammation and cell damage caused by viruses leads to dysfunction of the Eustachian tube, decreased ventilation of the middle ear and accumulation of fluid and nasopharyngeal bacteria into the middle ear cavity. Increased nasopharyngeal load of pneumococci is associated with both viral infection and PncAOM [10, 28–30]. Experimental studies have demonstrated synergistic associations between influenza A virus and pneumococci in development of PncAOM [27, 31–33]. The highest PncAOM incidence occurred when animals were challenged with pneumococci a few days after challenge with influenza, just before the time of influenza-induced leukocyte dysfunction and negative ear pressure [33]. These data support our finding that PncAOM emerges more frequently if pneumococcal acquisition occurs within the temporal vicinity of respiratory infection.

The PncAOM hazard was smallest if the child had been previously sick during the same long carriage. This agrees with our finding that preceding homologous carriage was associated with a lower PncAOM hazard. It is possible that interactions between pneumococci and respiratory viruses or repeated carriage acquisition stimulate development of specific immunity earlier or more effectively than what happens with maturation of immunity with age.

## Conclusions

In summary, we have presented data to argue that pneumococcal acquisition in temporal association with the acute phase of respiratory infection poses the highest risk of PncAOM. Because the currently available conjugate vaccines are thought to reduce acquisition rather than duration of carriage, our finding explains how the vaccine impact on carriage contributes to protection against PncAOM. Moreover, as the vaccine efficacy against both the overall vaccine-type acquisition and PncAOM is known to be only moderate [34,35], vaccine-induced protection against AOM is likely to act mostly through protection against carriage [36].

## Supporting Information

S1 Appendix Statistical modelling and analysis. (PDF)

S1 Fig Selection of data for defining episodes of carriage and non-carriage, sick episodes and risk episodes for pneumococcal acute otitis media in Finnish children followed from 2 months to 2 years of age 1994–97. NPS, nasopharyngeal swab; NPA, nasopharyngeal aspirate; MEF, middle ear fluid; PncAOM, pneumococcal acute otitis media. ^1)^Altogether 6 NPS and 7 NPA samples negative for pneumococci were imputed with positive carriage because the child was on antibiotic medication and the previous and subsequent samples were of the same serotype. In addition, in 5 occasions a negative NPA (2), missing NPA (2), or NPA with a different serotype from that in MEF (1) was imputed with the serotype isolated from the concurrent positive MEF sample. After these imputations the information of serotype was available for 1782 sick visits. In the 52 cases where 2 (or once 3) serotypes were identified concurrently, the serotype not present in the previous sample less than 62 days apart was chosen 35 times, the serotype in MEF was chosen 2 times, and in 15 cases the serotype was chosen randomly. Finally, the 439 risk episodes represented overlapping periods of 358 episodes of pneumococcal carriage and 382 sick episodes in 171 children.
(TIF)

S2 Fig Acquisition of pneumococcal carriage and respiratory infection (sensitivity analysis). This sensitivity analysis corresponds to Fig 3A in Section 3.4. However, episodes of carriage and non-carriage were censored if there was ≥45 days since the child’s previous carriage sample (as compared to 62 days in the base-case analysis). The figure presents the Nelson-Aalen estimate of the cumulative hazard of pneumococcal acquisition in the 286 children who had least 1 sick episode. For each sick episode, the at-risk time started at earliest at the end of the preceding sick episode onset and lasted until the end of the sick episode. The time origin is at the sick episode onset. Times of acquisition events were identified by the times of carriage episode onset if known by the episode definition with the 45 days rule.
(TIF)

S1 Table Hazard of pneumococcal otitis media by age group during risk episodes for which carriage had started more than 1 month before the sick episode onset. (DOC)
