# Supplementary material for: New Pneumococcal Carriage Acquired in Association with Acute Respiratory Infection Is Prone to Cause Otitis Media
Source: PLoS One. 2016 Jun 3;11(6):e0156343. doi: 10.1371/journal.pone.0156343 (PMC4892487; doi:10.1371/journal.pone.0156343)
Supplement: S1 Appendix — (PDF) [file pone.0156343.s001.pdf]

S1 Appendix. Statistical modelling and analysis

1. Markov transition model of pneumococcal acquisition and clearance

Here we specify in detail the Markov transition model that was used to estimate the hazards of pneumococcal acquisition and clearance from all age-based and sick visit samples in the FinOM Cohort Study.

For child i, let $\zeta_i(\tau), \tau \geq 0$, be a continuous-time Markov process with state space $\{0, 1, ..., n\}$, where 0 denotes non-carrier (susceptible) and $1, \dots, n$ denote the n ($= 30$) different serotypes/groups in the dataset. At any age $\tau > 0$, transitions between the $n+1$ states are governed by a transition rate matrix $\mathbf{\Lambda}_i(\tau) \in \mathbb{R}_+{}^{(n+1)\times(n+1)}$ with elements (transition rates or hazards):

$$\lambda_{j,k}^{(i)}(\tau) = \lim_{\Delta t \to 0} \frac{\mathbb{P}(\zeta_i(\tau + \Delta t) = k \mid \zeta_i(\tau) = j)}{\Delta t},$$

where $j, k \in \{0, ..., n\}$, $j \neq k$, and $\lambda_{k,k}^{(i)}(\tau) = -\sum_{j=0, j\neq k}^{n} \lambda_{k,j}^{(i)}(\tau)$.

To reduce the number of model parameters, the transition rates were parameterised as follows. For a non-carrying child i, the rates of acquisition were taken to be equal across all serotypes, i.e. $\lambda_{0,k}^{(i)}(\tau) = \lambda(\tau, S_i)$ for all $k \in \{1, \dots, n\}$. Here S_i is the observed information about the sick episodes of child i. In particular, if the child had M_i sick episodes, $S_i = \{[a_{im}, b_{im}]; m = 1, \dots, M_i\}$ and the acquisition rates were modelled as

$$\lambda_{0,k}^{(i)}(\tau) = \lambda(\tau, S_i) = \begin{cases} \phi_{pre}\lambda_j, & \text{if } \tau \in [a_{im} - \delta, a_{im}[\text{ for any } m = 1, \dots, M_i, \\ \phi_{dur}\lambda_j, & \text{if } \tau \in [a_{im}, b_{im}] \text{ for any } m = 1, \dots, M_i, \\ \lambda_j, & \text{otherwise,} \end{cases}$$

where δ is the duration of the pre-sickness period (30 days) and the baseline rate of acquisition $\lambda_j = \lambda_1$ (if age at the onset of sick episode $a_{im} < 12$ months)

or $\lambda_j = \lambda_2$ (if age $a_{im} \geq 12$ months). The parameters ϕ_{pre} and ϕ_{dur} are the relative rates of acquisition during the pre-sickness and sickness periods.

For a carrying child, the acquisition rates were multiplied by a "competition parameter" θ, i.e. $\lambda_{j,k}^{(i)}(\tau) = \theta\lambda_{0,k}^{(i)}(\tau)$ for all $j, k \in \{1, \ldots, n\}$, $k \neq j$. In addition, the clearance rates were defined as $\lambda_{k,0}(\tau) = \mu_1$, if $\tau < 12$ months, and μ_2, if $\tau \geq 12$ months, for all $k = 1, \ldots, n$. There were altogether 7 model parameters: $(\lambda_1, \lambda_2, \mu_1, \mu_2, \phi_{pre}, \phi_{dur}, \theta)$. The competition parameter θ was given a fixed value of 0.50 [1]. The six other parameters were estimated (cf. Table 3 in the main text) in the Bayesian setting, based on the likelihood function as explained in the next section.

2. Likelihood function and estimation of the transition rates

Denote the observed serotype/group-specific carriage status of child i at time τ_{ij} as $y_{ij} = \zeta_i(\tau_{ij})$, $i = 1, \ldots, N(= 329)$, $j = 1, \ldots, K_i$, where K_i is the number of observations of child i. These observations include both the age-based and sick visits samples. The child's status at birth (age $\tau_{i1} = 0$) is taken to be 0 (non-carrier). The carriage status was not usually observed, i.e. it was missing at the onset of the presickness period (ages $a_{im} - \delta$) and at the end of the sick episode (ages b_{im}). Denote the complete data, including both the observed and unobserved carriage statuses as $\{\tilde{y}_{ij}\}$ and the corresponding ages as $\{\tilde{\tau}_{ij}\}$. If the observation was missing at any time $\tilde{\tau}_{ik}$, denote $\tilde{y}_{ik} = *$.

Based on the observed data $\{y_{ij}; i = 1, \ldots, N, j = 1, \ldots, K_i\}$, the likelihood function of the six model parameters is

$$L(\lambda_1, \lambda_2, \mu_1, \mu_2, \phi_{pre}, \phi_{dur}; \{y_{ij}\}) = \prod_{i=1}^{N} \prod_{j=2}^{K_i} \sum_{s=1}^{n} \sum_{k=1}^{n} \mathbb{P}_{s,k}^{t(i,j)}(\mathbf{\Lambda_i}(\tau_{ij}))\mathbf{1}\{\tilde{y}_{ij} = k, \tilde{y}_{i,j-1} = s\},$$

where the lengths of the time intervals between two consecutive observations are $t(i, j) = \tau_{ij} - \tau_{i,j-1}$. The first term on the right-hand-side of the above expression is the transition probability for individual i having moved from

state s to state k during the time interval $]\tau_{i,j-1}, \tau_{ij}[$. It is computed as the (s,k)th element of the matrix exponential $\exp(t\boldsymbol{\Lambda}_i(\tau_{ij}))$. The second term on the right hand side is an indicator function that chooses the appropriate transition probability corresponding to the child's observations $\tilde{y}_{ij}$ and $\tilde{y}_{i,j-1}$. If the observation is missing (e.g. $\tilde{y}_{ij} = *$ for some k), the condition in the indicator function is void and the summation extends over all $n+1$ possible states of the process at time $\tilde{\tau}_{ij}$.

Because of the many symmetries (common parameters) in the model formulation, likelihood computations could be implemented using a 3-state model for successive carriage states: 0 (no carriage), carriage of any one of the types, carriage of another (i.e. any other) type. In particular, the states in an aggregated 3-state model were susceptible, carrier of one target type, carrier of any of the other types. For example, for a healthy child in the first age class, the transition rate matrix between the 3 states reads as follows:

$$\bar{\Lambda} = \begin{pmatrix} -n\lambda_1 & \lambda_1 & (n-1)\lambda_1 \\ \mu_1 & -\mu_1 - \theta_1(n-1)\lambda_1 & \theta_1(n-1)\lambda_1 \\ \mu_1 & \theta_1\lambda_1 & -\mu_1 - \theta_1\lambda_1 \end{pmatrix}$$

The transition probabilities required in the likelihood function can now be read from the elements of $\exp(t\bar{\Lambda})$. For example, the transition probability from a (carrier) state s to any other (carrier) state k ($\neq s$) is the (2,3)th element of the matrix exponent $\exp(t\bar{\Lambda})$, divided by $n-1$.

The likelihood function was implemented in statistical software R. Uninformative (flat) priors were defined for all model parameters. The posterior of the parameters was estimated using a tailored Markov Chain Monte Carlo (MCMC) algorithm, written in R. The implementation of the estimation routine and the identifiability of the model parameters were validated by simulated data. The results in Table 3 (main text) are based on 1800 MCMC samples. Because of the small number of parameters and the use of a marginal likelihood expression, the MCMC algorithm converged well. Parameter estimates are presented

as marginal posterior means and equitail 90% probability intervals.

Reference:

1. Hoti F, Erasto P, Leino T, Auranen K. Outbreaks of Streptococcus pneumoniae carriage in day care cohorts in Finland - implications for elimination of transmission. BMC Infect Dis. 2009;9:102,2334-9-102.
