# Supplementary figures and images for: New Pneumococcal Carriage Acquired in Association with Acute Respiratory Infection Is Prone to Cause Otitis Media
Source: PLoS One. 2016 Jun 3;11(6):e0156343. doi: 10.1371/journal.pone.0156343 (PMC4892487; doi:10.1371/journal.pone.0156343)

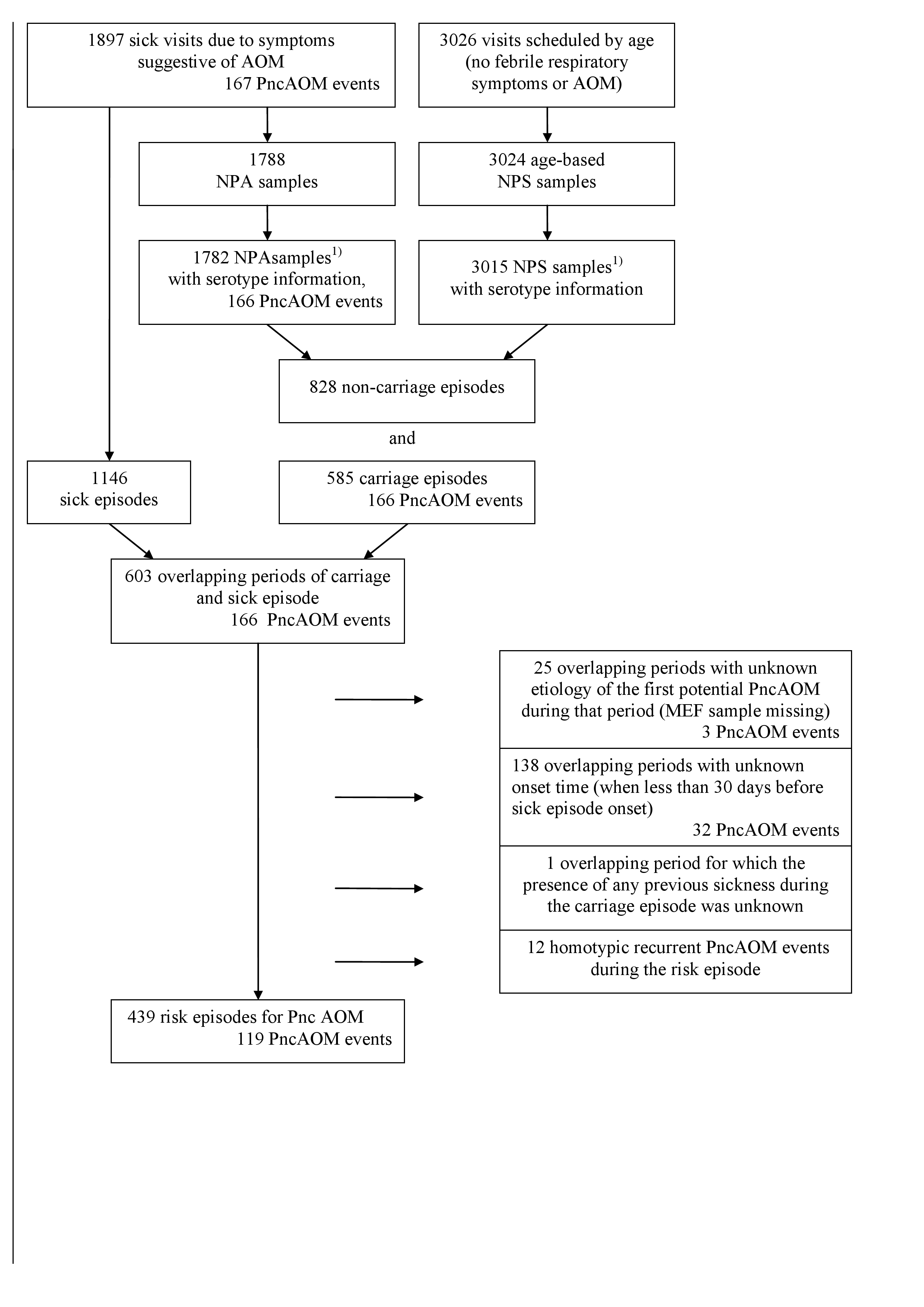

Supplement: S1 Fig — NPS, nasopharyngeal swab; NPA, nasopharyngeal aspirate; MEF, middle ear fluid; PncAOM, pneumococcal acute otitis media. 1)Altogether 6 NPS and 7 NPA samples negative for pneumococci were imputed with positive carriage because the child was on antibiotic medication and the previous and subsequent samples were of the same serotype. In addition, in 5 occasions a negative NPA (2), missing NPA (2), or NPA with a different serotype from that in MEF (1) was imputed with the serotype isolated from the concurrent positive MEF sample. After these imputations the information of serotype was available for 1782 sick visits. In the 52 cases where 2 (or once 3) serotypes were identified concurrently, the serotype not present in the previous sample less than 62 days apart was chosen 35 times, the serotype in MEF was chosen 2 times, and in 15 cases the serotype was chosen randomly. Finally, the 439 risk episodes represented overlapping periods of 358 episodes of pneumococcal carriage and 382 sick episodes in 171 children. (TIF) [file pone.0156343.s002.tif]

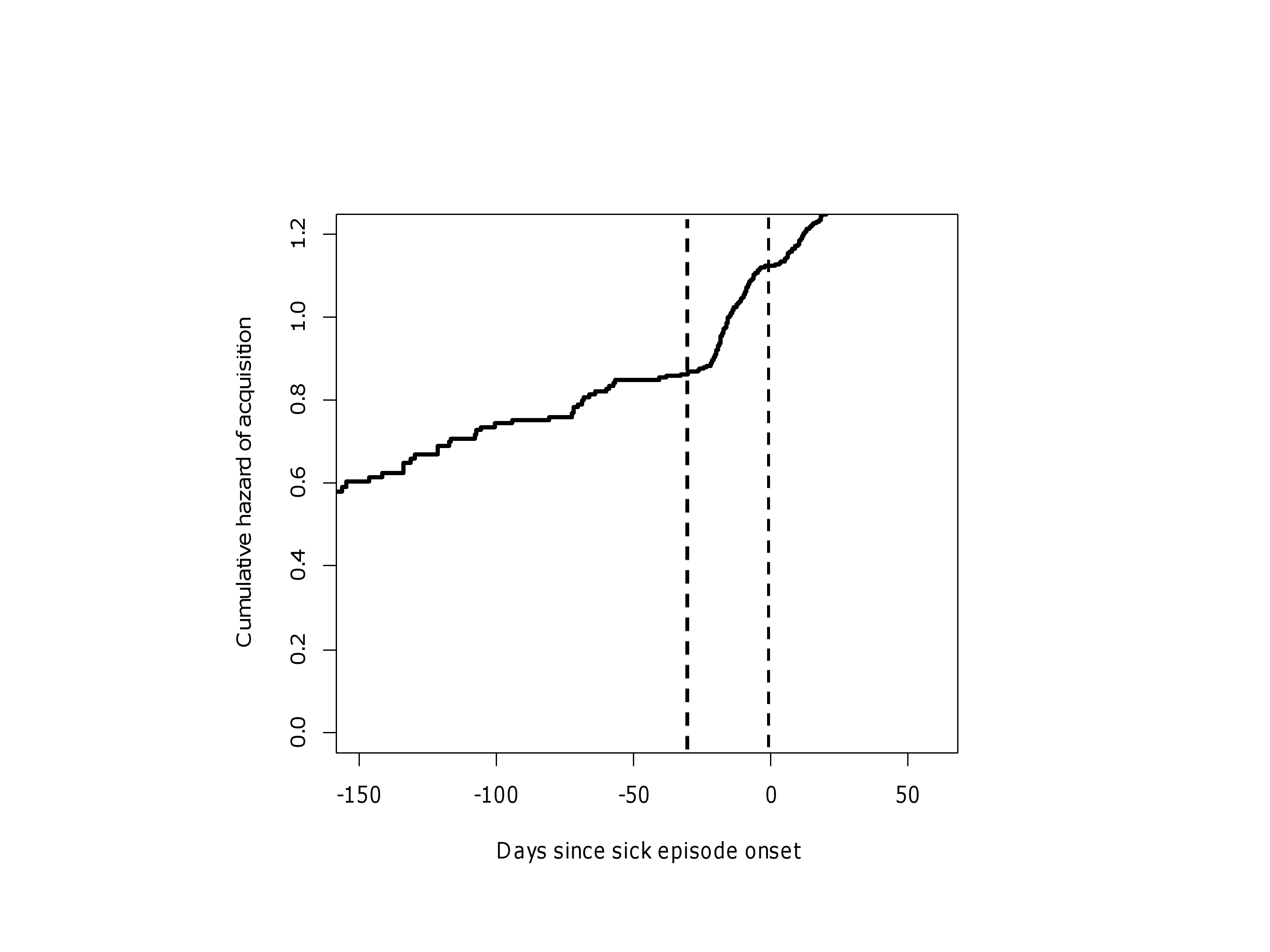

Supplement: S2 Fig — This sensitivity analysis corresponds to Fig 3A in Section 3.4. However, episodes of carriage and non-carriage were censored if there was ≥45 days since the child’s previous carriage sample (as compared to 62 days in the base-case analysis). The figure presents the Nelson-Aalen estimate of the cumulative hazard of pneumococcal acquisition in the 286 children who had least 1 sick episode. For each sick episode, the at-risk time started at earliest at the end of the preceding sick episode onset and lasted until the end of the sick episode. The time origin is at the sick episode onset. Times of acquisition events were identified by the times of carriage episode onset if known by the episode definition with the 45 days rule. (TIF) [file pone.0156343.s003.tif]
