# Supplementary material for: New Pneumococcal Carriage Acquired in Association with Acute Respiratory Infection Is Prone to Cause Otitis Media
Source: PLoS One. 2016 Jun 3;11(6):e0156343. doi: 10.1371/journal.pone.0156343 (PMC4892487; doi:10.1371/journal.pone.0156343)
Supplement: S1 Table — (DOC) [file pone.0156343.s004.doc]

S1 Table. Hazard of Pneumococcal Otitis Media by Age Group During Risk Episodes for Which Carriage Had Started More Than 1 Month Before the Sick Episode Onset, FinOM Cohort Study, Finland, 1994-1997.

|  | **Previous sick episode during the same underlying carriage episode^a^** | | | | | |
| --- | --- | --- | --- | --- | --- | --- |
| **Age group^b^** | **No** |  | **Yes** |  | **Total** |  |
| **(months)** | *N Y* $\hat{\lambda}$**^c^** 90% CI |  | *N Y* $\hat{\lambda}$ **^c^** 90% CI |  | *N Y* $\hat{\lambda}$**^c^** 90% CI | |
|  | (mo) (1/mo) (1/mo) |  | (mo) (1/mo) (1/mo) |  | (mo) (1/mo) (1/mo) | |
| <6 | 4 2.63 1.52 0.67,1.67 |  | 0 2.83 0.00 NA |  | 4 5.45 0.73 0.32,1.67 | |
| 6-11 | 5 7.90 0.63 0.30,1.32 |  | 8 15.16 0.53 0.29,0.94 |  | 13 23.06 0.56 0.36,0.89 | |
| 12-17 | 1 1.91 0.52 0.10,2.72 |  | 2 16.59 0.12 0.04,0.39 |  | 3 18.50 0.16 0.06,0.42 | |
| ≥18 | 0 0.34 0.00 NA |  | 3 3.73 0.80 0.31,2.08 |  | 3 4.07 0.74 0.28,1.90 | |
| Total | 10 12.78 0.78 0.47,1.32 |  | 13 38.31 0.34 0.22,0.54 |  | 23 51.09 0.45 0.32,0.63 | |

Abbreviations: CI, Bayesian posterior probability (credible) interval; mo, months; *N*, number of PncAOM events; NA, not available; PncAOM, pneumococcal acute otitis media; *Y*, person-time at risk during the risk episodes (overlapping periods of carriage and sickness, months).

**^a^** The hazards of PncAOM were estimated during risk episodes for which carriage had started more than 1 month before the sick episode onset. The data are stratified according to whether or not there were sick episodes during the same carriage episode.

**^b^** Age group at the onset of the risk episode.**^c^** Crude estimates of the hazard of PncAOM, $\lambda^{\wedge}$ (per month), were calculated as the number of PncAOM events (*N*) divided by the corresponding person-time (*Y*, months).
